# Vitamin B12, folate, and homocysteine levels in children and adolescents with obesity: a systematic review and meta-analysis

**DOI:** 10.3389/fpubh.2025.1481002

**Published:** 2025-02-07

**Authors:** Juan R. Ulloque-Badaracco, Esteban A. Alarcon-Braga, Enrique A. Hernandez-Bustamante, Beatrix M. Von-Koeller-Jones, Miguel Huayta-Cortez, Esduardo Saavedra-Custodio, Percy Herrera-Añazco, Vicente A. Benites-Zapata

**Affiliations:** ^1^Facultad de Ciencias de la Salud, Universidad Peruana de Ciencias Aplicadas, Lima, Peru; ^2^Sociedad Científica de Estudiantes de Medicina de la Universidad Nacional de Trujillo, Trujillo, Peru; ^3^Grupo Peruano de Investigación Epidemiológica, Unidad Para la Generación y Síntesis de Evidencias en Salud, Universidad San Ignacio de Loyola, Lima, Peru; ^4^Facultad de Ciencias de la Salud, Universidad Privada del Norte, Trujillo, Peru; ^5^Unidad de Investigación Para la Generación y Síntesis de Evidencias en Salud, Vicerrectorado de Investigación, Universidad San Ignacio de Loyola, Lima, Peru

**Keywords:** obesity, vitamin B12, folate, homocysteine, meta-analysis

## Abstract

**Background and aims:**

Childhood and adolescent obesity is a global public health concern. Obesity induces several metabolic disturbances. Several studies have explored the association of vitamin B12, folate, and homocysteine (Hcy) with obesity. This study aimed to synthesize the available evidence regarding the differences in serum levels of vitamin B12, Hcy, and folate among children or adolescents with and without obesity.

**Methods:**

A random-effects meta-analysis using the Sidik-Jonkman method and corrected 95% confidence interval (CI) using the truncated Knapp-Hartung standard errors was used for all meta-analyses. Standardized mean difference (SMD) with the corresponding 95% CI was used as the only effect size. The Cochran’s Q test and the I^2^ statistic were used to evaluate between-study heterogeneity. Publication bias was assessed using funnel plots and the Egger test.

**Results:**

Twenty studies were included with a combined study population of 7,791 patients. There were no significant differences between children/adolescents with and without obesity with respect to serum vitamin B12 levels (SMD: −0.24; 95% CI: −0.53 to 0.06; *p* > 0.05, I^2^ = 74.93%) and folate levels (SMD: −0.12; 95% CI: −0.29 to 0.06; *p* > 0.05, I^2^ = 19.6%). However, children/adolescents with obesity had significantly higher Hcy levels compared to counterparts without obesity (SMD: 0.77; 95% CI: 0.39 to 1.14; *p* < 0.001, I^2^ = 86.4%).

**Conclusion:**

Children and adolescents with obesity had higher Hcy levels than those without obesity. However, no significant differences were found for vitamin B12 and folate levels. Hcy may play a role in the development of obesity in this population.

## 1 Introduction

According to the World Health Organization (WHO), obesity in children and adolescents aged 5–19 years is defined as a body mass index (BMI) greater than 2 standard deviations above the WHO Growth Reference median (1). Childhood and adolescent obesity is a global public health concern (2, 3). According to research published jointly by UNICEF, WHO, and the World Bank in April 2019, globally, the prevalence of overweight for children under the age of 5 years increased from 4.8% in 1990 to 5.9% in 2018 (4). However, there is considerable heterogeneity regarding the estimates for low-and middle-income United Nations regions. The obesogenic environment has led to a modest global increase in obesity among children, primarily due to a lack of sufficient political will and interventions (4). Fueled by economic growth and lifestyle changes, obesity has emerged as an important health risk due to its long-term complications and earlier onset of chronic illnesses, such as cardiovascular disease, hypertension, and type 2 diabetes mellitus (5, 6).

Due to several metabolic disturbances induced by obesity, studies have explored the association between organic compounds (such as vitamin D and iron) and obesity (7, 8), with special emphasis on the triad of vitamin B12, folate, and homocysteine (Hcy) (9, 10). Hcy is an amino acid that results from the metabolism of methionine to cysteine (11), controlled by mutations on regulating enzymes (9) that use vitamin B12 and folate as cofactors in the remethylation pathway (10). In this context, several studies have identified Hcy as a nontraditional marker of obesity, due to the ability to release mediators of inflammation in patients with obesity and cause endothelial damage (11).

In parallel, the essential vitamins B12 and folate play a pivotal role in numerous physiological processes, including the synthesis of DNA and the production of red blood cells. Children and adolescents with obesity have higher rates of chronic inflammatory diseases, which can interfere with the metabolism of B12 and folate through the release of pro-inflammatory cytokines (12). In consequence, they are more exposed to lifelong use of medications, which would explain the increased risk of B12 and folate deficiency (13).

Some pre-clinical studies have suggested a key role of B12 deficiency in obesity by inducing adipogenesis and cholesterol synthesis (14, 15). In patients with obesity, adipose tissue serves as an active endocrine organ that leads to the release of leptin, adiponectin, and resistin. These adipokines can impact the metabolism of homocysteine and B vitamins, resulting in changes in their levels (16). It is important to highlight that although scientific evidence supports these hypotheses, there is considerable inter-individual variability in this respect (17, 18). Diets that are high in processed foods and low in essential nutrients can lead to deficiencies of B12 and folate (17). Coexisting pathologies, anthropometric parameters, and changes in gut microbiota can also cause disruptions in their absorption and bioavailability (18).

Although diverse studies have identified associations between obesity and the above-mentioned organic compounds, the results are inconclusive (19, 20), especially in relation to their levels in children with obesity. Therefore, there is a lack of consensus in this field of study (21). The objective of this study was to conduct a systematic review and meta-analysis to synthesize the existing evidence on the differences in serum levels of vitamin B12, Hcy, and folate among children or adolescents with and without obesity.

## 2 Methods

### 2.1 Registration and reporting

We provided a condensed form of the protocol for systematic reviews, adhering to the International Prospective Register of Systematic Reviews (PROSPERO) [CRD42023402162]. The results are reported according to the Preferred Reporting Items for Systematic Reviews and Meta-Analyses (PRISMA) statement guidelines (22).

### 2.2 Search strategy and databases

The PRESS Checklist was used to build the search strategy, which was later adapted for all databases (23). No limitations were imposed in terms of date or language of publication. On February 13, 2023, a simultaneous systematic search was performed across PubMed, Scopus, Embase, LILACS, Ovid-Medline, and Web of Science databases. Furthermore, the reference lists of the included studies and preprint databases were manually screened. The full search strategy is provided in Supplementary Table S1. The research question was based on the Population, Exposure, Comparison, and Outcome (PECO) strategy: Do children and adolescents (P) with obesity (E) have lower levels of vitamin B12, folate, and homocysteine (O) compared to children and adolescents without obesity (C)?

The research question was based on Population (Children and adolescents), Exposure (Obesity), Comparison (no obesity), and Outcome (Vitamin B12, folate, and Hcy) strategy.

### 2.3 Study selection and data extraction

Cross-sectional, case–control, or cohort studies that assessed the association between vitamin B12, folate, or Hcy levels with obesity in children/adolescents were eligible for inclusion. The exclusion criteria were as follows: duplicate publications, conference abstracts, scoping reviews, systematic reviews, randomized controlled trial (RCT), and narrative reviews. Among the mentioned, RCT were excluded because they are intervention studies that aim to assess a diseased population; whereas observational studies allow us to study the levels of vitamin B12, folate and Hcy in both healthy and diseased populations.

The articles retrieved from the databases were transferred to a software for data management called Rayyan © (24). After the elimination of duplicates, four authors individually screened the titles and abstracts of all articles against the selection criteria. Subsequently, full texts of the short-listed articles were independently reviewed by two authors. Any article that did not meet the entire set of selection criteria was excluded from the review. For articles with missing information, the authors were contacted. Any conflict of opinion or discrepancy in any of the phases was resolved by consensus.

The selection process was depicted using the PRISMA flowchart. A standardized data collection sheet developed in Google Sheets© was used for data extraction. Data pertaining to the following variables were independently extracted by two authors: first author, study location, publication date, study design, sample size, age and sex distribution of the study population, definition of obesity, number of patients with obesity, vitamin B12 levels in patients with and without obesity, folate levels in patients with and without obesity, Hcy levels in patients with and without obesity, and assay technique. Obesity was defined based on the parameters established by each author of the included articles. The definitions of obesity are presented in Supplementary Table S2.

### 2.4 Risk of bias and publication bias

The risk of bias was evaluated independently by two authors. For cohort and case–control studies, the Newcastle-Ottawa Scale (NOS) was employed, while an adaptation of the NOS for cross-sectional studies (NOS-CS) was used for cross-sectional studies. The NOS assesses the methodological quality (risk of bias) of a study based on three main aspects: the selection of study groups, the comparability of the groups, and the assessment of outcomes or exposures. The NOS consists of eight items (seven items if it is the NOS-CS), with each item scored up to one star, except for comparability, which can be scored up to two stars. A score of ≥7 stars was regarded as a low risk of bias (high methodological quality), while a score of <7 stars was deemed a high risk of bias (low methodological quality). The effect of potential publication bias on the results of the meta-analysis was evaluated using funnel plots, the Egger test, and the trim-and-fill method (25). For funnel plots, the minimum number of studies required was 10.

### 2.5 Data synthesis

Statistical analysis was performed using STATA 17.0©. Standardized mean difference (SMD) with the corresponding 95% confidence interval (CI) was used as the only effect size. Median values and their interquartile ranges were converted into mean and the corresponding standard deviation (SD) using Hozo’s method (26). For variables with the standard errors (SE) reported, SD was determined using the following equation: SE × √ (sample size). In addition, the natural logarithm of the odds ratio (lnOR) and its standard error were transformed into SMD and its 95% CI using Chinn’s method (27). A random-effects meta-analysis was performed using the Sidik-Jonkman method and corrected 95%CI using the truncated Knapp-Hartung standard error (28, 29). The Cochran’s Q test and the I^2^ statistic were employed to evaluate between-study heterogeneity; high heterogeneity was defined as I^2^ ≥ 60% and a *p*-value <0.05. Subgroup analyses were performed by study design and assay method. A sensitivity analysis was conducted according to risk of bias and obesity definition.

## 3 Results

### 3.1 Study selection

**Figure 1:**
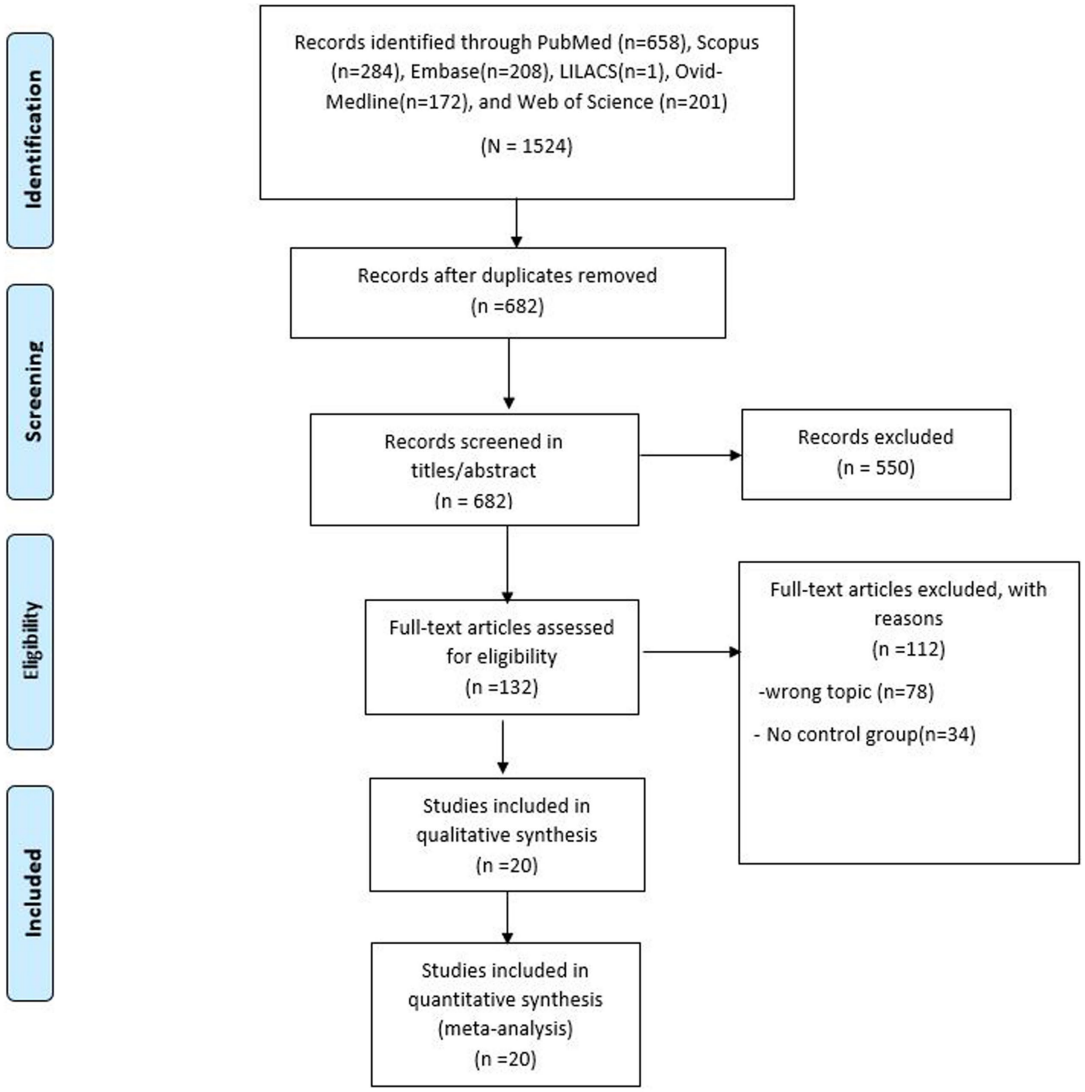
PRISMA flow diagram.

The systematic literature search yielded 1,524 records; after the elimination of duplicates, 682 records remained. After the screening of titles/abstracts, 132 articles were selected for full-text review. Finally, only 20 records complied with all the selection criteria and were included in this systematic review (30–49). The selection process is summarized in the PRISMA flow diagram (Figure 1).

### 3.2 Characteristics of included studies

The characteristics of the included studies are summarized in Table 1. A total of 20 studies (5 case–control, 14 cross-sectional, and one cohort study) that assessed the association of vitamin B12, folate, and Hcy levels with obesity in children and adolescents were included. These studies were conducted between 2004 and 2022 in various countries: Turkey (6 studies), India (3 studies), Spain (2 studies), Israel (2 studies), United States (1 study), South Korea (1 study), Egypt (1 study), China (1 study), Brazil (1 study), Romania (1 study), and Tunisia (1 study). The combined total study population in the 20 studies was 7,791. Only 12 studies (*n* = 4,066) reported the number of male (*n* = 1834) and female (*n* = 2,232) participants included.

**Table 1:** Characteristics of the included studies.

Author	Year	Study design	Country	Median/mean/Range age (IQR/SD)	Participants (male/female)	Marker analyzed	Marker mean (SD) in patients with obesity	Marker mean (SD) in patients without obesity	Assay method
Narin F et al.	2005	Case–Control	Turkey	Obese: 11.4 (2.5) Control: 11.6 (2.6)	60 (34/26)	Hcy	14.3 (11.8)	8.7 (5.9)	HPLC
						Folate	10.7 (5.1)	10.8 (8.5)	RIA
						Vitamin B12	602.3 (283.8)	592.2 (314.5)	RIA
Pinhas-Hamiel O et al.	2006	Cohort	Israel	<12	206 (NR/NR)	Vitamin B12	494.75 (145.92)	554 (210.37)	CLIA
Gunanti I et al.	2014	Cross-sectional	United States of America/ Mexico	8–15	821(NR/NR)	Folate	12.9 (0.27)	13.4 (0.2)	RIA
						Vitamin B12	534 (11.6)	613 (8.56)	RIA
Atabek M et al. (A)	2004	Cross-sectional	Turkey	Obese: 12.9 (2.2) Control: 13.5 (1.9)	38 (20/18)	Hcy	6.1 (1.8)	4.6 (1.1)	CLIA
						Folate	9.62 (3.08)	9.2 (4.3)	CLIA
						Vitamin B12	271.5 (90.89)	230.7 (49.7)	CLIA
Chakraborty S et al.	2018	Cross-sectional	India	14 (12–15)	2,403 (1,024/1379)	Vitamin B12	NR	NR	ECLIA
Ozer S et al.	2017	Case–Control	Turkey	Obese: 12.69 (2.29) Control: 13.05 (2.48)	256 (95/161)	Vitamin B12	298.25 (112.97)	351.15 (149.61)	NR
Yoon J et al.	2006	Case–Control	South Korea	8–11	57 (31/26)	Hcy	8.1 (2.1)	4.9 (1)	FPI
						Folate	9.8 (3.7)	9.8 (3.5)	FPI
						Vitamin B12	798.6 (174.3)	967.8 (405)	FPI
Kassem E et al.	2022	Cross-sectional	Israel	11.3 (0.5)	125 (NR/NR)	Folate	6.7 (4.2)	7.99 (3.2)	ECLIA
						Vitamin B12	485 (163.5)	527.9 (202.5)	ECLIA
Awasthi S et al.	2021	Cross-sectional	India	6–16	2,276 (NR/NR)	Folate	NR	NR	CLIA
						Vitamin B12	NR	NR	CLIA
Abaci A et al.	2012	Cross-sectional	Turkey	Obese:10.2 (2.7) Control: 10.9 (2.6)	170 (NR/NR)	Hcy	10.8 (5)	10.8 (5)	ELISA
Kandil M et al.	2011	Cross-sectional	Egypt	Obese: 9.25 (2.63) Control: 10.07 (2.28)	82 (43/39)	Hcy	11.49 (3.8)	9.44 (2.24)	EIA
Codoñer-Franch P et al.	2014	Cross-sectional	Spain	Obese: 12 (8–13) Control: 11 (8–13)	110 (63/47)	Hcy	7.75 (1.33)	6.525 (0.815)	ECLIA
Huang X et al.	2005	Cross-sectional	China	Obese: 10.8 (2.3) Control: 10.9 (2)	94 (67/27)	Hcy	7.9 (2.7)	5.6 (2.1)	CLIA
Da Silva N et al.	2013	Cross-sectional	Brazil	8.9 (6.5–11.5)	677 (330/347)	Hcy	NR	NR	HPLC
Atabek M et al. (B)	2007	Cross-sectional	Turkey	11.7 (2.5)	100 (50/50)	Hcy	9.8 (3.9)	8.3 (3.6)	CLIA
Ezgü F et al.	2009	Cross-sectional	Turkey	6–9	58 (NR/NR)	Hcy	5.07 (2.89)	5.04 (2.12)	HPLC
Kumar K et al.	2017	Cross-sectional	India	5–15	98 (NR/NR)	Hcy	17.225 (6.741)	7.65 (3.704)	HPLC
Dimitriu L et al.	2014	Cross-sectional	Romania	8–18	21 (NR/NR)	Hcy	8.98 (1.73)	6.1 (2.4)	CLIA
Gara S et al.	2011	Case–Control	Tunisia	4–14	53 (NR/NR)	Hcy	10.34 (4.86)	11 (4.26)	FPI
						Folate	5.75 (5.9)	5.4 (6.08)	RIA
						Vitamin B12	676.3 (519.1)	902.6 (544.8)	RIA
Martos R et al.	2006	Case–Control	Spain	6–9	86 (34/52)	Hcy	6.82 (0.15)	6.4 (0.18)	FPI

NR, not reported; Hcy, homocysteine; CLIA, chemiluminescence immunoassay; ECLIA, electrochemiluminescence immunoassay; ELISA, enzyme-linked immune-sorbent assay; FPI, fluorescence polarization immunoassay; HPLC, high-performance liquid chromatography; RIA, radio immunoassay.

The methods used to measure Hcy, vitamin B12, and folate levels were Chemiluminescence Immunoassay (CLIA), Electrochemiluminescence Immunoassay (ECLIA), Enzyme-linked Immunosorbent Assay (ELISA), Fluorescence Polarization Immunoassay (FPI), High-Performance Liquid Chromatography (HPLC), Radioimmunoassay (RIA), and Enzyme immunoassays (EIA).

### 3.3 Risk of bias assessment

The risk of bias assessment of the included studies using the NOS and the NOS-CS revealed that 3 studies had a high risk of bias (low methodological quality) while the remaining 17 articles had a low risk of bias (high methodological quality; Supplementary Table S3).

### 3.4 Differences in serum vitamin B12 levels among children or adolescents with and without obesity

**Figure 2:**
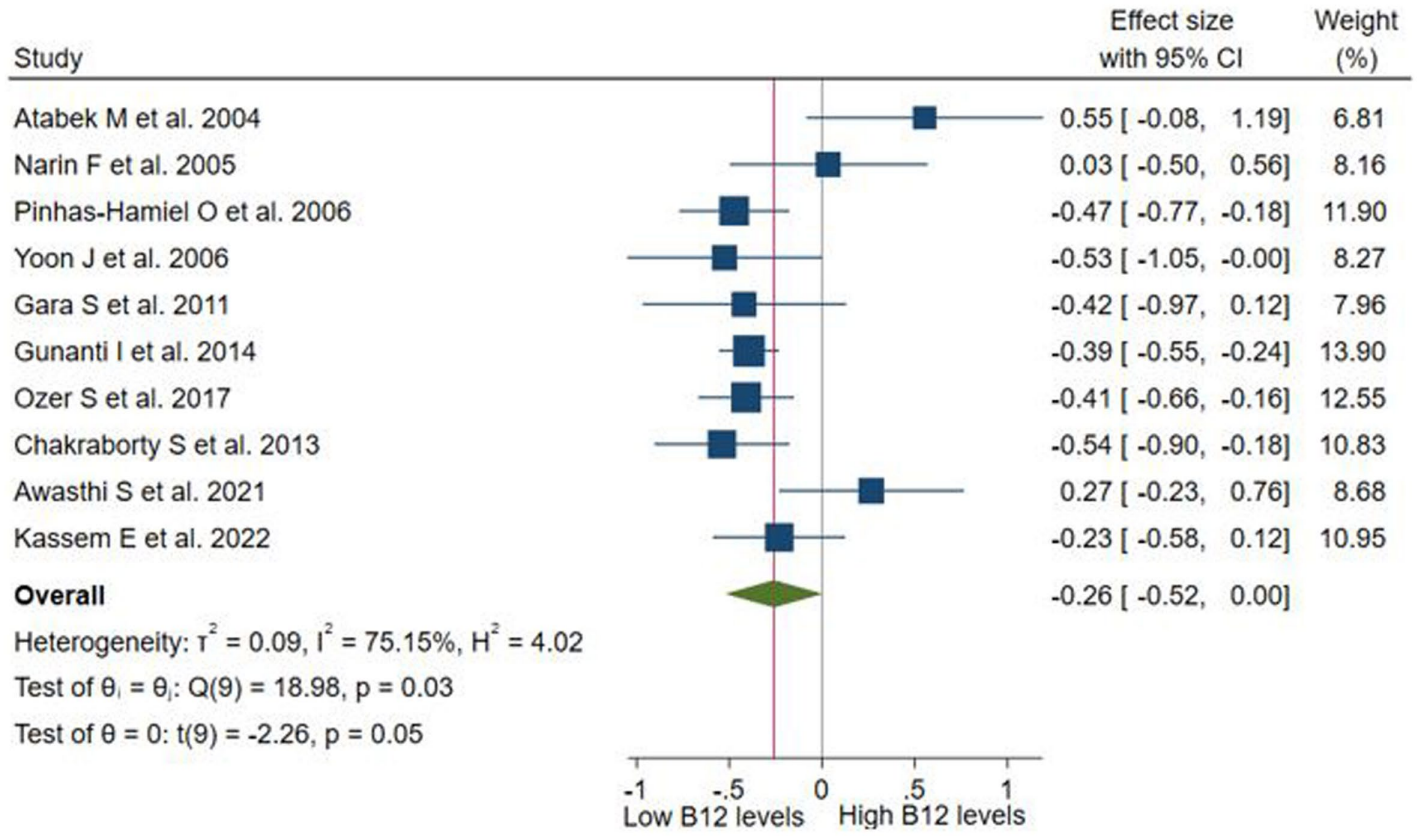
Differences in serum levels of vitamin B12 among children or adolescents with and without obesity.

These differences were evaluated in 10 studies (n = 6,295). No significant difference was observed in the vitamin B12 levels between children/adolescents with and without obesity (SMD: −0.26; 95% CI: −0.52 to 0.00; *p* > 0.05, I^2^ = 75.15%; Figure 2). Subgroup analyses performed according to study design (Supplementary Figure S1), assay method (Supplementary Figure S2), and continents (Supplementary Figure S3) revealed no significant differences in these subgroups. No high heterogeneity (I^2^ ≥ 60%) was found in the subgroups that evaluated B12 using RIA or in case–control studies. In the sensitivity analysis according to risk of bias (Supplementary Figure S4), the lack of significant difference and high heterogeneity was maintained (SMD: −0.26; 95% CI: −0.59 to 0.07; *p* > 0.05, I^2^ = 73.97%).

The funnel plot (Supplementary Figure S5) and the Egger test (*p* < 0.1) indicated potential publication bias, which was corrected by the trim-and-fill method (SMD = −0.42, 95% CI −0.69 to −0.14; Supplementary Figure S6).

### 3.5 Differences in serum folate levels among children or adolescents with and without obesity

**Figure 3:**
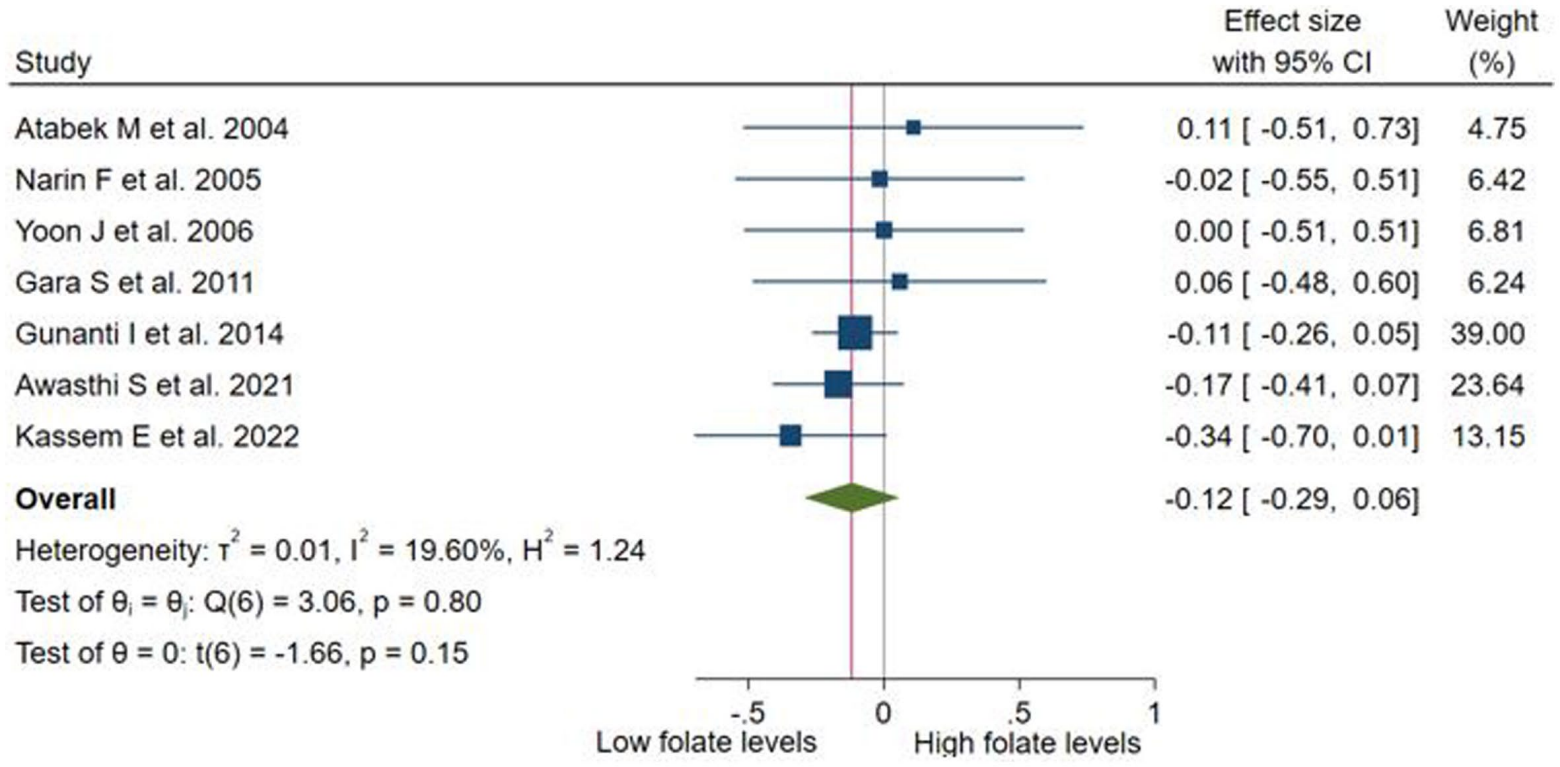
Differences in serum levels of folate among children or adolescents with and without obesity.

These differences were evaluated in 07 studies (*n* = 3,430). No significant difference was observed in the folate levels between children/adolescents with and without obesity (SMD: −0.12; 95% CI: −0.29 to 0.06; *p* > 0.05, I^2^ = 19.6%; Figure 3). Subgroup analyses performed according to study design (Supplementary Figure S7), assay method (Supplementary Figure S8), and continents (Supplementary Figure S9) revealed no significant differences in these subgroups. No high heterogeneity (I^2^ ≥ 60%) was found in the subgroups that evaluated folate using RIA, as well as in case–control studies, cross-sectional studies, and studies conducted in Asia. In the sensitivity analysis according to risk of bias (Supplementary Figure S10), the lack of significant difference was maintained (SMD: −0.13; 95% CI: −0.42 to 0.15; *p* > 0.05, I^2^ = 21.97%). The Egger test indicated no significant publication bias (*p* = 0.48).

### 3.6 Differences in serum Hcy levels among children or adolescents with and without obesity

**Figure 4:**
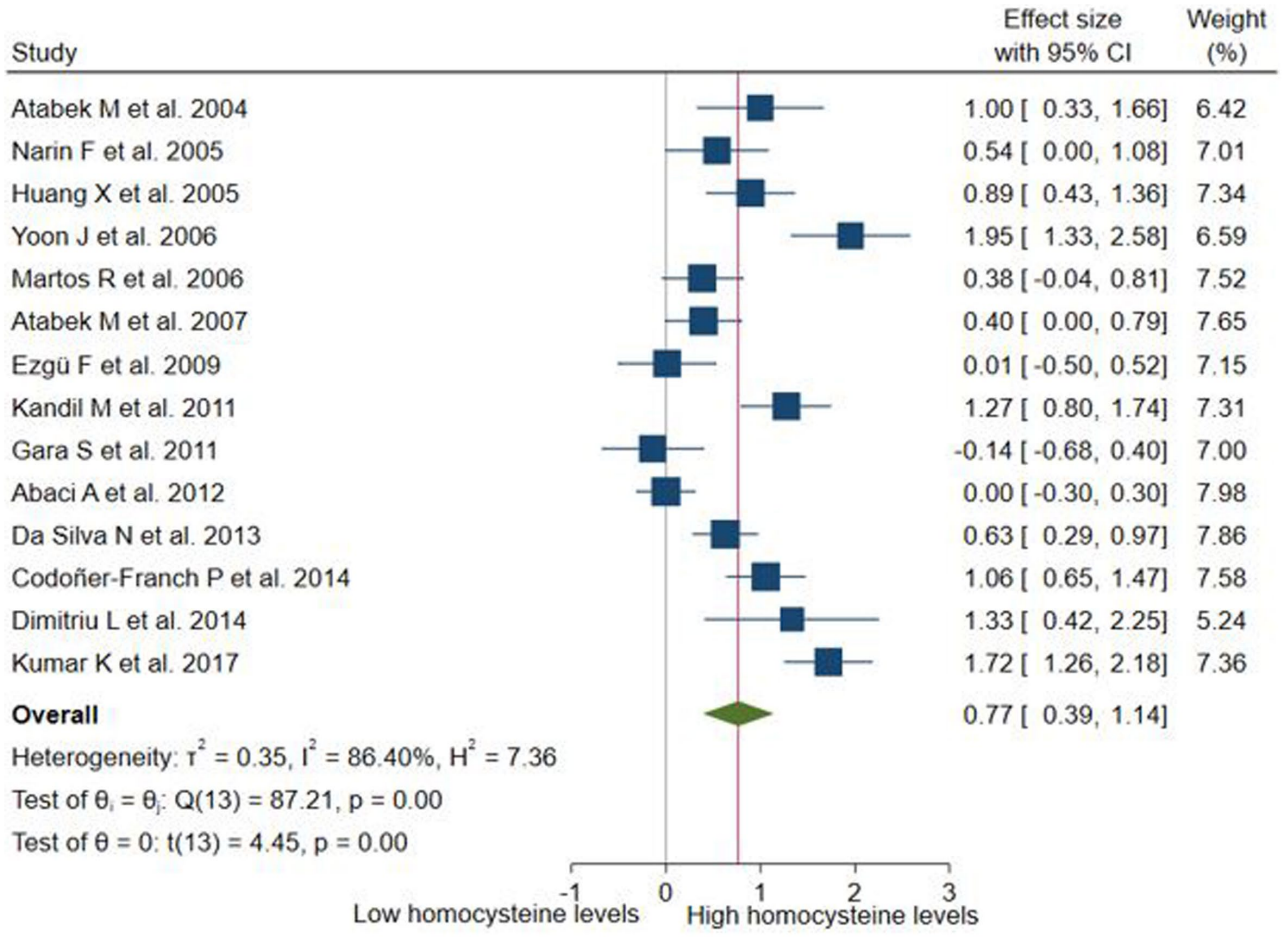
Differences in serum levels of homocysteine among children or adolescents with and without obesity.

These differences were evaluated in 14 studies (*n* = 1704). Children and adolescents with obesity had significantly higher Hcy levels than those without obesity (SMD: 0.77; 95% CI: 0.39 to 1.14; *p* < 0.001, I^2^ = 86.4%; Figure 4). Subgroup analyses were performed according to study design (Supplementary Figure S11), assay method (Supplementary Figure S12), and continents (Supplementary Figure S13). Significant differences were only observed in the subgroups that evaluated Hcy with CLIA, cross-sectional studies, and studies conducted in Asia. The only subgroup that did not exhibit high heterogeneity was the one that evaluated Hcy using CLIA. The sensitivity analysis according to risk of bias (Supplementary Figure S14) maintained a significant difference and high heterogeneity (SMD: 0.86; 95% CI: 0.44 to 1.27; *p* < 0.001, I^2^ = 85.5%). Similarly, in the sensitivity analysis according to the definition of obesity (Supplementary Figure S15), the significant difference and high heterogeneity were maintained (SMD: 0.65; 95% CI: 0.19 to 1.12; *p* < 0.05, I^2^ = 87.58%), despite the exclusion of studies that did not report the definition for obesity (42, 46, 47), as well as the study (49) that defined obesity as a BMI > 90th percentile, due to the risk of including overweight participants. The funnel plot (Supplementary Figure S16) and the Egger test revealed no significant publication bias (*p* = 0.13).

## 4 Discussion

The key finding of this study was that children and adolescents with obesity had higher Hcy levels than their counterparts without obesity. This difference was sustained in the sensitivity and some subgroup analyses. Of note, there were no significant differences in vitamin B12 and folate levels among children or adolescents with and without obesity. The associations between obesity with folate, and homocysteine did not demonstrate publication bias. However, the association between vitamin B12 and obesity did show publication bias, which, when corrected using the trim-and-fill method, revealed a significant difference.

A previous systematic review has shown an association between Hcy levels and obesity in children and adolescents (21). However, to the best of our knowledge, there are no systematic reviews on the differences between their levels in children and adolescents with and without obesity. Another systematic review found that adult patients with obesity had significantly higher levels of Hcy compared to their counterparts without obesity, using standardized mean difference as a measure of effect size (9).

Hcy is a naturally occurring amino acid in the human body, formed during the metabolism of methionine to cysteine (50), derived from dietary proteins. Variations in its levels are widely known to affect various chronic conditions, particularly cardiovascular disorders (51, 52). Even though the relationship between high Hcy levels and obesity is multifaceted, significant correlations have been identified by researchers with several potential underlying mechanisms. First, due to poor eating habits and reduced nutrient absorption (53), individuals with obesity are more prone to deficiencies of vitamins that participate in Hcy metabolism, such as B6, B12, and folate. These vitamins act as cofactors during the remethylating pathway of Hcy (54), contributing to elevated Hcy levels in the context of poor-quality food intake. Second, due to the inadequate diet, individuals with obesity often display alterations in gut microbiota (55). Certain gut bacteria interfere with the body’s ability to metabolize Hcy, thus elevating circulating levels (56). Third, obesity is associated with impaired insulin sensitivity (57), a condition where cells become unresponsive to insulin. Even though the relationship is not entirely clear, some studies suggest that insulin resistance can affect the regulation of Hcy metabolism (58), and therefore also contribute to hyperhomocysteinemia. Lastly, inflammation plays a key role in Hcy levels in subjects with obesity (59). The chronic release of pro-inflammatory cytokines (60, 61), combined with obesity-related alterations in gut microbiota (62), contribute both to systemic inflammation and the production of metabolites that affect the body’s ability to break down Hcy, potentially elevating its circulating levels.

Nevertheless, there is considerable heterogeneity regarding the differences in serum Hcy among children or adolescents with and without obesity. This may be attributable to the differences in population characteristics, geographical variability, confounding factors, and statistical approaches. Indeed, a study has shown the differential geographical distribution of the risk variants in the folate/Hcy metabolic pathway relative to ethnic background in Mexico (63).

Our study does not attempt to explain the reasons for the lack of differences in the levels of vitamin B12 and folate in children and adolescents with and without obesity. However, it is possible to propose some hypotheses related to the complex relationship between these vitamins and obesity. For example, the relationship between folate and obesity is not straightforward; low folate levels have been linked in certain studies to an increased risk of obesity or weight gain because folate deficiency may change how energy is metabolized, resulting in a gradual weight increase (64). As for vitamin B12, there are indirect mechanisms by which it may be linked to weight management (31). On one hand, the metabolism of proteins, lipids, and carbohydrates depends heavily on vitamin B12 (65). It has been argued that increasing B12 levels may enhance energy metabolism; however, there is little empirical evidence that B12 supplementation alone can cause considerable weight loss (66). On the other hand, studies have also implied that B12 plays a role in appetite-regulating hormones and its deficiency can lead to anemia and exhaustion, thus indirectly affecting physical activity levels and obesity (14). Likewise, although our study only evaluated differences in vitamin B12 and folate levels, the primary studies that were part of the analysis did not evaluate dietary habits. This is important because some studies have suggested a greater need for folic acid and vitamin B supplementation in certain areas of the world (63). Similarly, recent advances in the field of nutrigenetics have highlighted the impact of genetic variations on individuals’ responses to dietary intake. In the case of vitamin B-complex, special reference has been made to the widely studied variant in the 5,10-methylenetetrahydrofolate reductase (*MTHFR*) gene (66).

Serum folate and total Hcy levels are influenced by folate intake and genetic polymorphisms in the *MTHFR* gene such as C677T (67). The prevalence of the MTHFR 677TT genotype varies across ethnic groups and regions, with a frequency of approximately 15% in Japanese populations (67). These differences may also explain our findings because individuals with the TT genotype have significantly higher serum Hcy levels and lower serum folate levels than those with the CT and TT genotypes (67).

### 4.1 Limitations and strengths

This study has some limitations. First, differences with respect to the study population (e.g., age, sex, ethnicity, and underlying health conditions) and study characteristics might have led to substantial heterogeneity across the included studies. However, subgroup and sensitivity analyses were performed to explore potential sources of heterogeneity. Second, the pooled estimates were based on crude measures of effect size, which may have potentially been affected by non-controlled confounders. Third, although there is at least one included study per continent, the generalizability of our findings is limited. Fourth, the sensitivity and specificity of optimal cut-off levels of the biomarkers assessed have not been reported. Further studies should establish these cut-off levels to evaluate the prognostic value of these biomarkers in children and adolescents with obesity. Nonetheless, our study has several strengths. Multiple databases were searched, allowing a comprehensive synthesis of the available literature. A substantial number of participants were included, which ensured adequate statistical power.

## 5 Conclusion

In summary, children and adolescents with obesity had higher Hcy levels than their counterparts without obesity. However, no statistically significant differences were found for vitamin B12 and folate levels. These findings highlight the potential role of Hcy in the development of obesity in this population. Although further research is required to elucidate the underlying mechanisms and establish causality, these findings underscore the importance of early detection and targeted interventions to address elevated Hcy levels as a potential risk factor for obesity in the pediatric population. Future studies should explore the interaction between Hcy, nutritional status, and obesity to improve our understanding and inform preventive strategies in the context of childhood and adolescent obesity.

## Data Availability

The original contributions presented in the study are included in the article/Supplementary material, further inquiries can be directed to the corresponding author.
