# Supplementary material for: Vitamin B12, folate, and homocysteine levels in children and adolescents with obesity: a systematic review and meta-analysis
Source: Front Public Health. 2025 Feb 7;13:1481002. doi: 10.3389/fpubh.2025.1481002 (PMC11842448; doi:10.3389/fpubh.2025.1481002)

Supplementary Material

**Vitamin B12, folate, and homocysteine levels in children and adolescents with obesity: A systematic review and meta-analysis**

**Supplementary Table S1. Search Strategy**

| **Source** | **PubMed** |
| --- | --- |
| **Search** | **Formula** |
| **#1** | Obesity [MH] |
| **#2** | Child[MH] |
| **#3** | Vitamin B 12 [MH] OR (“vitamin*” [TIAB] AND “B12” [TIAB]) OR (“b” [TIAB] AND “12” [TIAB]) OR “cyanocobalamin*” [TIAB] OR “cobalamin*” [TIAB] OR “eritron*” [TIAB] |
| **#4** | Folic Acid [MH] OR ((“Folic” [TIAB] OR “Pteroylglutamic” [TIAB]) AND “Acid” [TIAB]) OR (“Vitamin” [TIAB] AND (“M” [TIAB] OR “B9” [TIAB])) OR “Folvite” [TIAB] OR “Folacin” [TIAB] OR “Folate” [TIAB] |
| **#5** | Homocysteine [MH] OR “2-amino-4-mercaptobutyric acid” [TIAB] OR “2 amino 4 mercaptobutyric acid” [TIAB] OR “homocysteine” [TIAB] OR “2 amino 4 mercaptobutyric acid” [TIAB] OR “betahomocystein” [TIAB] |
| **#6** | #3 OR #4 OR #5 |
| **#7** | #1 AND #2 AND #6 |
| **Source** | **Scopus** |
| **Search** | **Formula** |
| **#1** | TITLE-ABS-KEY(“Obesity*” ) |
| **#2** | TITLE-ABS-KEY(“Childhood*” ) |
| **#3** | TITLE-ABS-KEY ((“vitamin*” W/3 “B12”) OR (“b” W/3 “12”) OR “cyanocobalamin*” OR “cobalamin*” OR “eritron*”) |
| **#4** | TITLE-ABS-KEY(((“Folic” OR “Pteroylglutamic”) W/3 “Acid”) OR (“Vitamin” W/3 (“M” OR “B9”)) OR “Folvite” OR “Folacin” OR “Folate”) |
| **#5** | TITLE-ABS-KEY(“2-amino-4-mercaptobutyric acid” OR “2 amino 4 mercaptobutyric acid” OR “homocysteine” OR “2 amino 4 mercaptobutyric acid” OR “betahomocystein”) |
| **#6** | #3 OR #4 OR #5 |
| **#7** | #1 AND #2 AND #6 |
| **Source** | **Web of Science** |
| **Search** | **Formula** |
| **#1** | TI=(“Obesity*”) |
| **#2** | TI=(“ Child*”) |
| **#3** | TI=((“vitamin*” NEAR/3 “B12”) OR (“b” NEAR/3 “12”) OR “cyanocobalamin*” OR “cobalamin*” OR “eritron*”) OR AB=((“vitamin*” NEAR/3 “B12”) OR (“b” NEAR/3 “12”) OR “cyanocobalamin*” OR “cobalamin*” OR “eritron*”) OR AK=((“vitamin*” NEAR/3 “B12”) OR (“b” NEAR/3 “12”) OR “cyanocobalamin*” OR “cobalamin*” OR “eritron*”) OR KP=((“vitamin*” NEAR/3 “B12”) OR (“b” NEAR/3 “12”) OR “cyanocobalamin*” OR “cobalamin*” OR “eritron*”) OR TS=((“vitamin*” NEAR/3 “B12”) OR (“b” NEAR/3 “12”) OR “cyanocobalamin*” OR “cobalamin*” OR “eritron*”) |
| **#4** | TI=(((“Folic” OR “Pteroylglutamic”) NEAR/3 “Acid”) OR (“Vitamin” NEAR/3 (“M” OR “B9”)) OR “Folvite” OR “Folacin” OR “Folate”) OR AB=(((“Folic” OR “Pteroylglutamic”) NEAR/3 “Acid”) OR (“Vitamin” NEAR/3 (“M” OR “B9”)) OR “Folvite” OR “Folacin” OR “Folate”) OR KP=(((“Folic” OR “Pteroylglutamic”) NEAR/3 “Acid”) OR (“Vitamin” NEAR/3 (“M” OR “B9”)) OR “Folvite” OR “Folacin” OR “Folate”) OR AK=(((“Folic” OR “Pteroylglutamic”) NEAR/3 “Acid”) OR (“Vitamin” NEAR/3 (“M” OR “B9”)) OR “Folvite” OR “Folacin” OR “Folate”) OR TS=(((“Folic” OR “Pteroylglutamic”) NEAR/3 “Acid”) OR (“Vitamin” NEAR/3 (“M” OR “B9”)) OR “Folvite” OR “Folacin” OR “Folate”) |
| **#5** | TI=(“2-amino-4-mercaptobutyric acid” OR “2 amino 4 mercaptobutyric acid” OR “homocysteine” OR “2 amino 4 mercaptobutyric acid” OR “betahomocystein”) OR AB=(“2-amino-4-mercaptobutyric acid” OR “2 amino 4 mercaptobutyric acid” OR “homocysteine” OR “2 amino 4 mercaptobutyric acid” OR “betahomocystein”) OR AK=(“2-amino-4-mercaptobutyric acid” OR “2 amino 4 mercaptobutyric acid” OR “homocysteine” OR “2 amino 4 mercaptobutyric acid” OR “betahomocystein”) OR KP=(“2-amino-4-mercaptobutyric acid” OR “2 amino 4 mercaptobutyric acid” OR “homocysteine” OR “2 amino 4 mercaptobutyric acid” OR “betahomocystein”) OR TS=(“2-amino-4-mercaptobutyric acid” OR “2 amino 4 mercaptobutyric acid” OR “homocysteine” OR “2 amino 4 mercaptobutyric acid” OR “betahomocystein”) |
| **#6** | #3 OR #4 OR #5 |
| **#7** | #1 AND #2 AND #6 |
| **Source** | **Embase** |
| **Search** | **Formula** |
| **#1** | ('Obesity/exp ti):kw |
| **#2** | ('Child/exp ti):kw |
| **#3** | 'cyanocobalamin'/exp OR ((“vitamin*” NEAR/3 “B12”) OR (“b” NEAR/3 “12”) OR “cyanocobalamin*” OR “cobalamin*” OR “eritron*”):ti OR ((“vitamin*” NEAR/3 “B12”) OR (“b” NEAR/3 “12”) OR “cyanocobalamin*” OR “cobalamin*” OR “eritron*”):ab OR ((“vitamin*” NEAR/3 “B12”) OR (“b” NEAR/3 “12”) OR “cyanocobalamin*” OR “cobalamin*” OR “eritron*”):kw |
| **#4** | 'folic acid'/exp OR (((“Folic” OR “Pteroylglutamic”) NEAR/3 “Acid”) OR (“Vitamin” NEAR/3 (“M” OR “B9”)) OR “Folvite” OR “Folacin” OR “Folate”):ti OR (((“Folic” OR “Pteroylglutamic”) NEAR/3 “Acid”) OR (“Vitamin” NEAR/3 (“M” OR “B9”)) OR “Folvite” OR “Folacin” OR “Folate”):ab OR (((“Folic” OR “Pteroylglutamic”) NEAR/3 “Acid”) OR (“Vitamin” NEAR/3 (“M” OR “B9”)) OR “Folvite” OR “Folacin” OR “Folate”):kw |
| **#5** | 'homocysteine'/exp OR (“2-amino-4-mercaptobutyric acid” OR “2 amino 4 mercaptobutyric acid” OR “homocysteine” OR “2 amino 4 mercaptobutyric acid” OR “betahomocystein”):ti OR (“2-amino-4-mercaptobutyric acid” OR “2 amino 4 mercaptobutyric acid” OR “homocysteine” OR “2 amino 4 mercaptobutyric acid” OR “betahomocystein”):ab OR (“2-amino-4-mercaptobutyric acid” OR “2 amino 4 mercaptobutyric acid” OR “homocysteine” OR “2 amino 4 mercaptobutyric acid” OR “betahomocystein”):kw |
| **#6** | #3 OR #4 OR #5 |
| **#7** | #1 AND #2 AND #5 |
| **Source** | **OVID** |
| **Search** | **Formula** |
| **#1** | ('Obesity*).kw. |
| **#2** | ('Child*).kw. |
| **#3** | ((vitamin* adj3 B12) OR (b adj3 12) OR cyanocobalamin* OR cobalamin* OR eritron*).ti. OR ((vitamin* adj3 B12) OR (b adj3 12) OR cyanocobalamin* OR cobalamin* OR eritron*).ab. OR ((vitamin* adj3 B12) OR (b adj3 12) OR cyanocobalamin* OR cobalamin* OR eritron*).kw. |
| **#4** | (((Folic OR Pteroylglutamic) adj3 Acid) OR (Vitamin adj3 (M OR B9)) OR Folvite OR Folacin OR Folate).ti. OR (((Folic OR Pteroylglutamic) adj3 Acid) OR (Vitamin adj3 (M OR B9)) OR Folvite OR Folacin OR Folate).ab. OR (((Folic OR Pteroylglutamic) adj3 Acid) OR (Vitamin adj3 (M OR B9)) OR Folvite OR Folacin OR Folate).kw. |
| **#5** | (2-amino-4-mercaptobutyric acid OR 2 amino 4 mercaptobutyric acid OR homocysteine OR 2 amino 4 mercaptobutyric acid OR betahomocystein).ti. OR (2-amino-4-mercaptobutyric acid OR 2 amino 4 mercaptobutyric acid OR homocysteine OR 2 amino 4 mercaptobutyric acid OR betahomocystein).ab. OR (2-amino-4-mercaptobutyric acid OR 2 amino 4 mercaptobutyric acid OR homocysteine OR 2 amino 4 mercaptobutyric acid OR betahomocystein).kw. |
| **#6** | #3 OR #4 OR #5 |
| **#7** | #1 AND #2 AND #6 |
| **Source** | **LILACS** |
| **Search** | **Formula** |
| **#1** | vitamin B12 [Palavras] OR folate [Palavras] OR homocysteine [Palavras] and obesity [palavras] |

AND: will narrow down a search; OR: Either of the Mesh Terms must appear in the results.

All terms used are based on MeSH (Medical Subject Headings) terms.

**Supplementary Table S2. Definitions for clinical diagnosis of obesity used in the included studies**

| Author | Definition |
| --- | --- |
| Narin F et al. | BMI>95th percentile for age and sex |
|  |  |
|  |  |
| Pinhas-Hamiel O et al. | BMI>95th percentile for age and sex |
| Gunanti I et al. | BMI>95th percentile for age and sex |
|  |  |
| Atabek M et al. (A) | BMI z-score > 2 standard deviations for age and sex. |
|  |  |
|  |  |
| Chakraborty S et al. | International Obesity Taskforce were adapted based on the extrapolation of adult BMI cut-off point for obesity (30 kg/m^2^) from children living in six countries |
| Ozer S et al. | BMI>95th percentile for age and sex |
| Yoon J et al. | Korean Pediatric Society 1998  Obesity (%) = (Current weight - Standard weight by height) / Standard weight by height × 100  Obesity >120% |
|  |  |
|  |  |
| Kassem E et al. | BMI z-score > 2 standard deviations for age and sex. |
|  |  |
| Awasthi S et al. | BMI z-score > 2 standard deviations for age and sex. |
|  |  |
| Abaci A et al. | BMI>95th percentile for age and sex |
| Kandil M et al. | BMI>95th percentile for age and sex |
| Codoñer-Franch P et al. | International Obesity Taskforce |
| Huang X et al. | NR |
| Da Silva N et al. | BMI>95th percentile for age and sex |
| Atabek M et al. (B) | BMI>95th percentile for age and sex |
| Ezgü F et al. | BMI>95th percentile for age and sex |
| Kumar K et al. | NR |
| Dimitriu L et al. | NR |
| Gara S et al. | BMI>97th percentile for age and sex |
|  |  |
|  |  |
| Martos R et al. | BMI>90th percentile for age and sex |

NR: Not reported

**Supplementary Table S3. Newcastle - Ottawa quality assessment scale for included studies**

| **NEWCASTLE - OTTAWA QUALITY ASSESSMENT SCALE FOR COHORT STUDIES** | | | | | | | | | | | |
| --- | --- | --- | --- | --- | --- | --- | --- | --- | --- | --- | --- |
| **STUDY** | **SELECTION** | | | | **COMPARABILITY** | **OUTCOME** | | |  |  | |
|  | **Representativeness of the exposed cohort** | **Selection of the non-exposed cohort** | **Ascertainment of exposure** | **Demonstration that outcome of interest was not present at start of study** | **Comparability of Cohorts on the Basis of the Design or Analysis Maximum : ☆☆** | **Assessment of outcome** | **Was follow-up long enough for outcomes to occur** | **Adequacy of follow up of cohorts** | **SCORE** | **Evidence quality** |  |
| Pinhas-Hamiel O et al. | **☆** | **☆** | **☆** | **☆** | **☆** | **☆** | **☆** | **☆** | *8* | Low Risk of Bias |  |

| **NEWCASTLE - OTTAWA QUALITY ASSESSMENT SCALE FOR CASE-CONTROL STUDIES** | | | | | | | | | | | | | | | | |
| --- | --- | --- | --- | --- | --- | --- | --- | --- | --- | --- | --- | --- | --- | --- | --- | --- |
| **STUDY** | | **SELECTION** | | | | | **COMPARABILITY** | | **EXPOSURE** | | | |  | |  | |
|  | | **Is the case definition adequate?** | | **Representativeness of the cases** | **Selection of Controls** | **Definition of Controls** | **Comparability of cases and controls on the basis of the design or analysis (Maximum : ☆☆ )** | | **Ascertainment of Exposure** | | **Same method of Ascertainment for Cases and Controls** | **Non-Response Rate** | **SCORE** | | **Evidence quality** | |
| Narin F et al. | | **☆** | | **☆** | **☆** | **☆** |  | | **☆** | | **☆** |  | 6 | | High Risk of Bias | |
| Ozer S et al. | | **☆** | | **☆** | **☆** | **☆** | **☆** | | **☆** | | **☆** | **☆** | 8 | | Low Risk of Bias | |
| Yoon J et al. | | **☆** | | **☆** | **☆** | **☆** | **☆** | | **☆** | | **☆** | **☆** | 8 | | Low Risk of Bias | |
| Gara S et al. | | **☆** | | **☆** | **☆** | **☆** | **☆** | | **☆** | | **☆** |  | 7 | | Low Risk of Bias | |
| Martos R et al. | | **☆** | | **☆** | **☆** | **☆** | **☆** | | **☆** | | **☆** | **☆** | 8 | | Low Risk of Bias | |

| **NEWCASTLE - OTTAWA QUALITY ASSESSMENT SCALE FOR CROSS-SECTIONAL STUDIES** | | | | | | | | | | | | | |
| --- | --- | --- | --- | --- | --- | --- | --- | --- | --- | --- | --- | --- | --- |
| **STUDY** | | **SELECTION** | | | | | **COMPARABILITY** | **OUTCOME** | | | |  | |
|  | | **Sample Size** | | **Representativeness of the cases** | **Non-Response Rate** | **Ascertainment of the screening/surveillance tool** | **The subjects in different outcome groups are comparable, based on the study design or analysis. Confounding factors are controlled.**  **Maximum : ☆☆** | | **Assessment of outcome**  **Maximum : ☆☆** | **Statistical Tests** | **SCORE** | **Evidence quality** | |
| Gunanti I et al. | | **☆** | | **☆** |  | **☆** | **☆** | | **☆** | **☆** | 6 | High Risk of Bias | |
| Atabek M et al. (A) | | **☆** | | **☆** | **☆** | **☆** | **☆** | | **☆** | **☆** | 7 | Low Risk of Bias | |
| Chakraborty S et al. | | **☆** | | **☆** | **☆** | **☆** | **☆☆** | | **☆** | **☆** | 8 | Low Risk of Bias | |
| Kassem E et al. | | **☆** | | **☆** | **☆** | **☆** | **☆☆** | | **☆** | **☆** | 8 | Low Risk of Bias | |
| Awasthi S et al. | | **☆** | | **☆** | **☆** | **☆** | **☆☆** | | **☆** | **☆** | 8 | Low Risk of Bias | |
| Abaci A et al. | | **☆** | | **☆** |  | **☆** | **☆** | | **☆** | **☆** | 6 | High Risk of Bias | |
| Kandil M et al. | | **☆** | | **☆** | **☆** | **☆** | **☆☆** | | **☆** | **☆** | 8 | Low Risk of Bias | |
| Codoñer-Franch P et al. | | **☆** | | **☆** | **☆** | **☆** | **☆☆** | | **☆** | **☆** | 8 | Low Risk of Bias | |
| Huang X et al. | | **☆** | | **☆** | **☆** | **☆** | **☆☆** | | **☆** | **☆** | 8 | Low Risk of Bias | |
| Da Silva N et al. | | **☆** | | **☆** | **☆** | **☆** | **☆☆** | | **☆** | **☆** | 8 | Low Risk of Bias | |
| Atabek M et al. (B) | | **☆** | | **☆** | **☆** | **☆** | **☆☆** | | **☆** | **☆** | 8 | Low Risk of Bias | |
| Ezgü F et al. | | **☆** | | **☆** | **☆** | **☆** | **☆☆** | | **☆** | **☆** | 8 | Low Risk of Bias | |
| Kumar K et al. | | **☆** | | **☆** | **☆** | **☆** | **☆☆** | | **☆** | **☆** | 8 | Low Risk of Bias | |
| Dimitriu L et al. | | **☆** | | **☆** | **☆** | **☆** | **☆☆** | | **☆** | **☆** | 8 | Low Risk of Bias | |

**Figure S1. Vitamin B12 and obesity in children/adolescents: Subgroup Analysis according to study design**

**
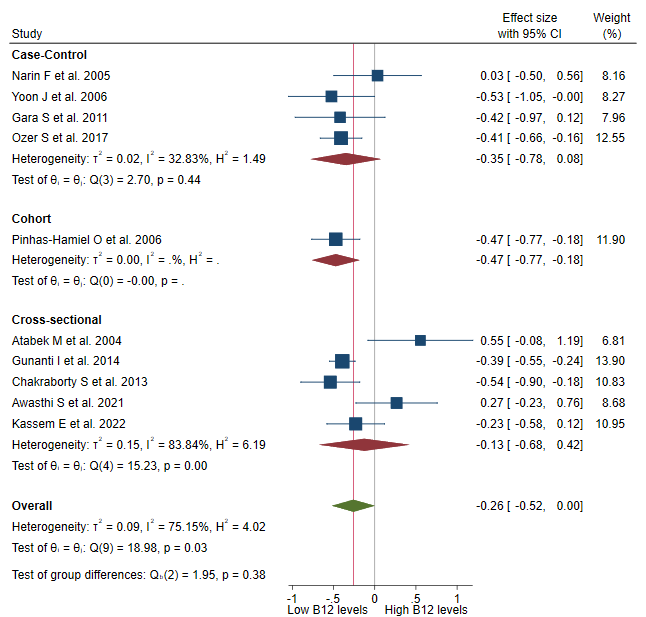
**

**Figure S2. Vitamin B12 and obesity in children/adolescents: Subgroup Analysis according to assay method**


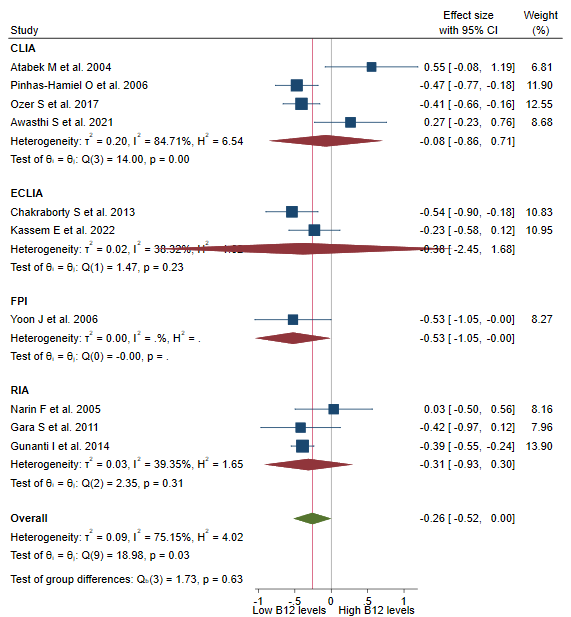


**Figure S3. Vitamin B12 and obesity in children/adolescents: Subgroup Analysis according to continents**

**
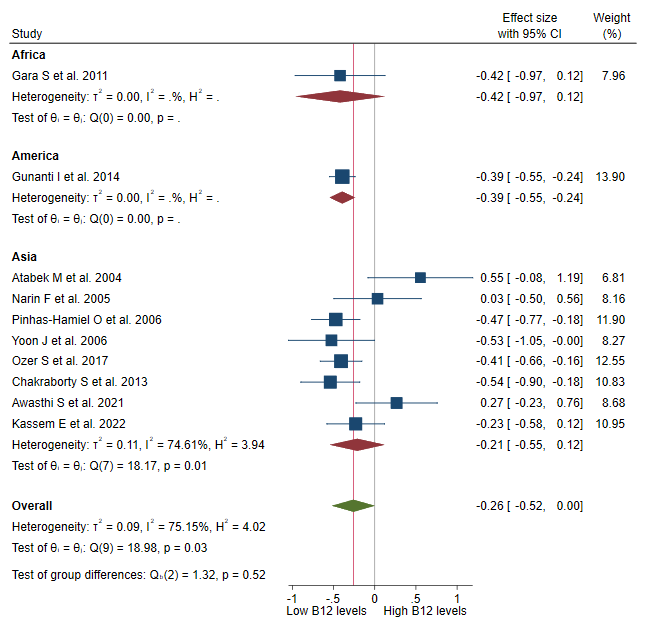
**

**Figure S4. Vitamin B12 and obesity in children/adolescents: Sensitivity analysis according to risk of bias**


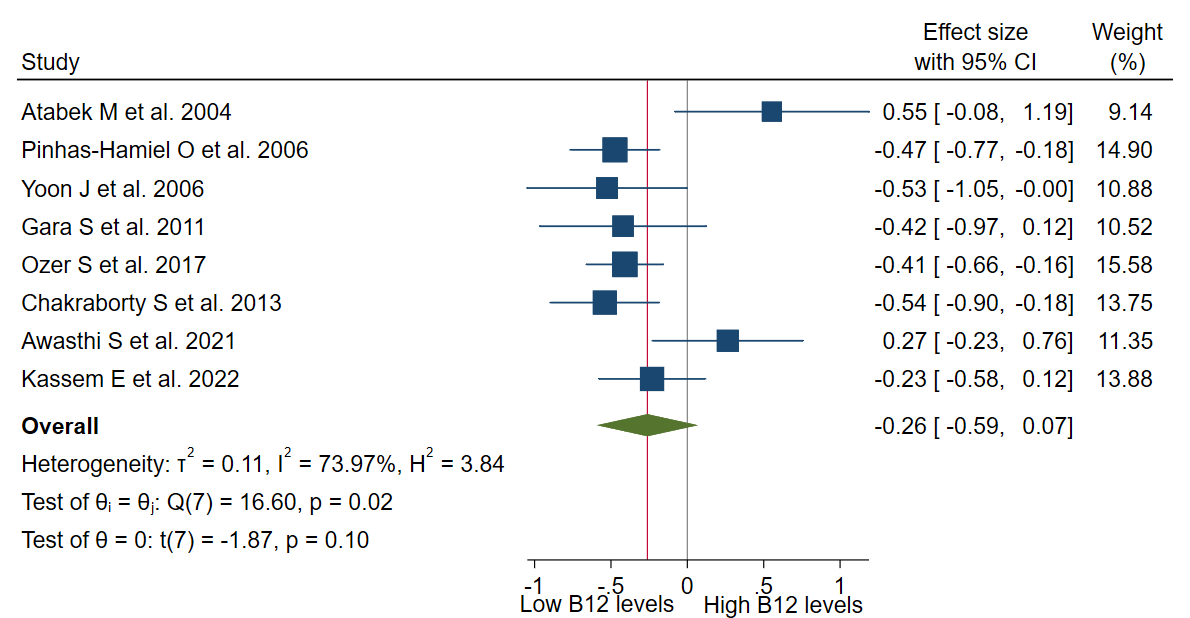


**Figure S5. Funnel Plot: Vitamin B12 and obesity in children/adolescents**


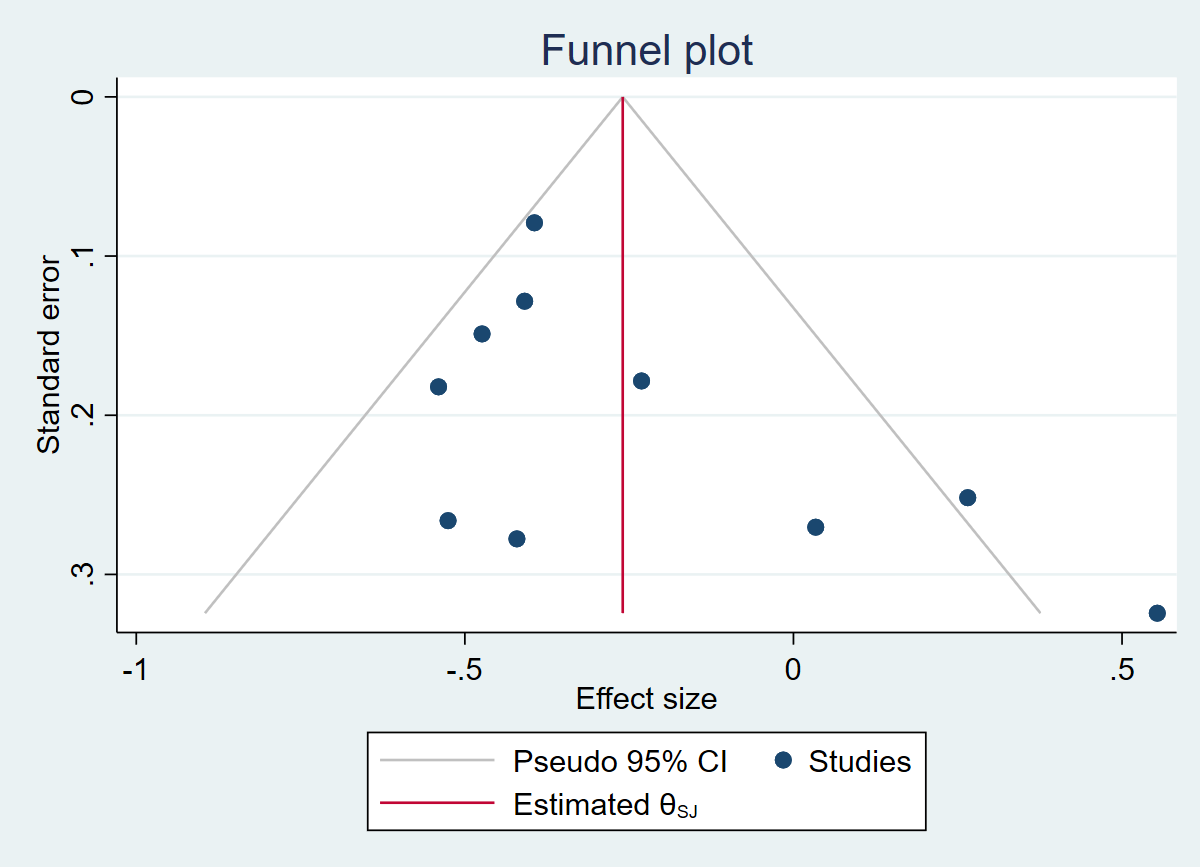


**Figure S6. Trim-and-fill method: Vitamin B12 and obesity in children/adolescents**

**
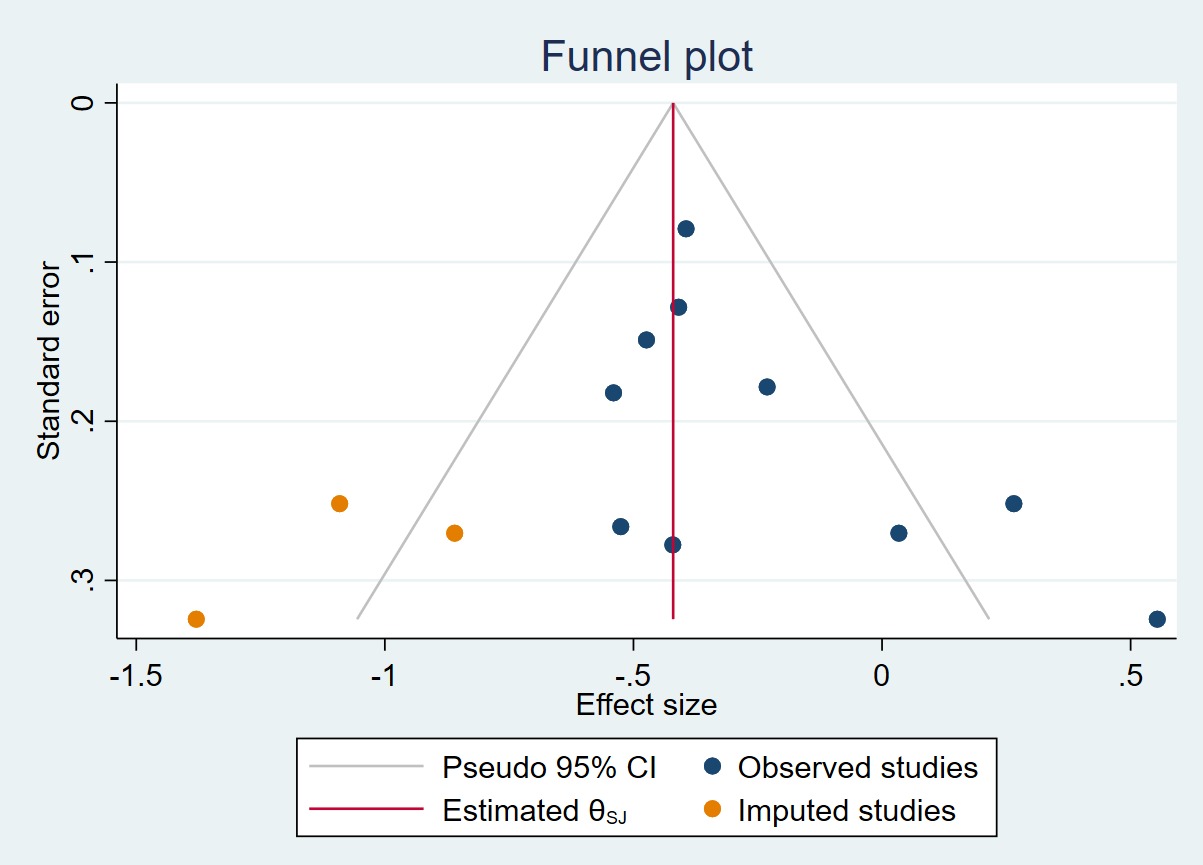
**

**Figure S7. Folate and obesity in children/adolescents: Subgroup Analysis according to study design**


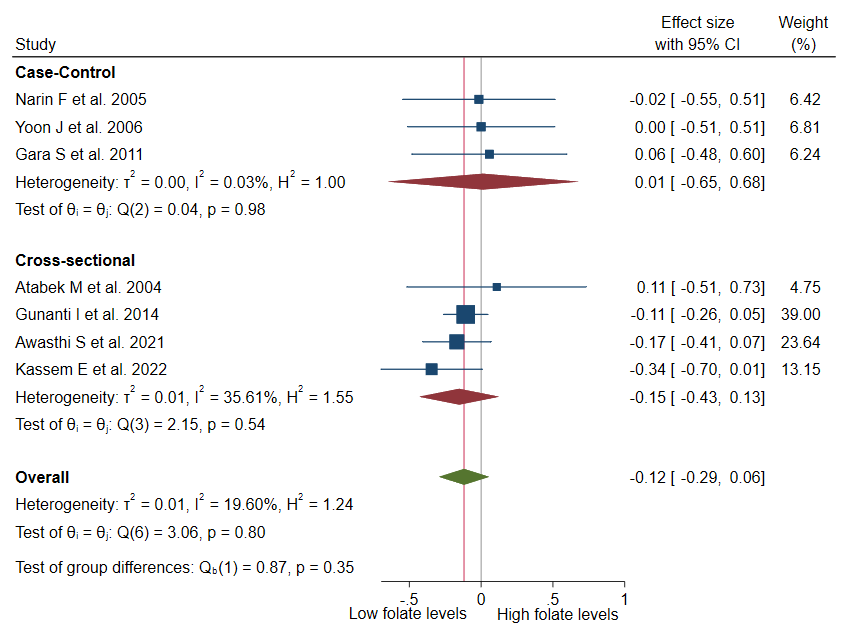


**Figure S8. Folate and obesity in children/adolescents: Subgroup Analysis according to assay method**

**
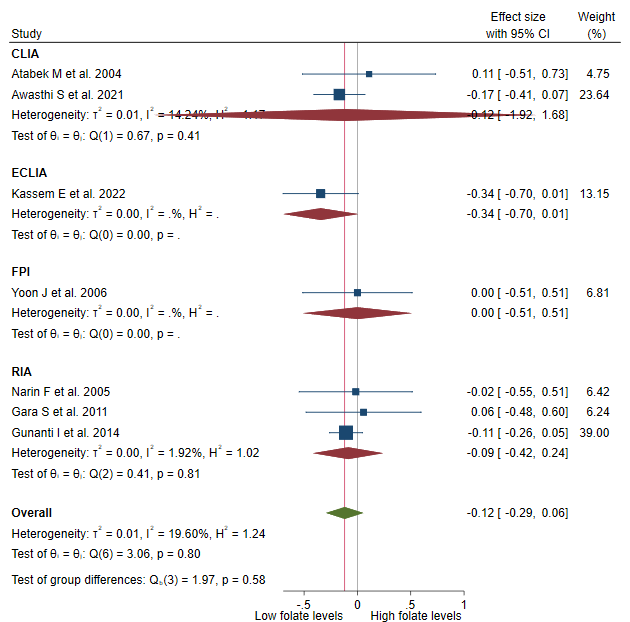
**

**Figure S9. Folate and obesity in children/adolescents: Subgroup Analysis according to continents**

**
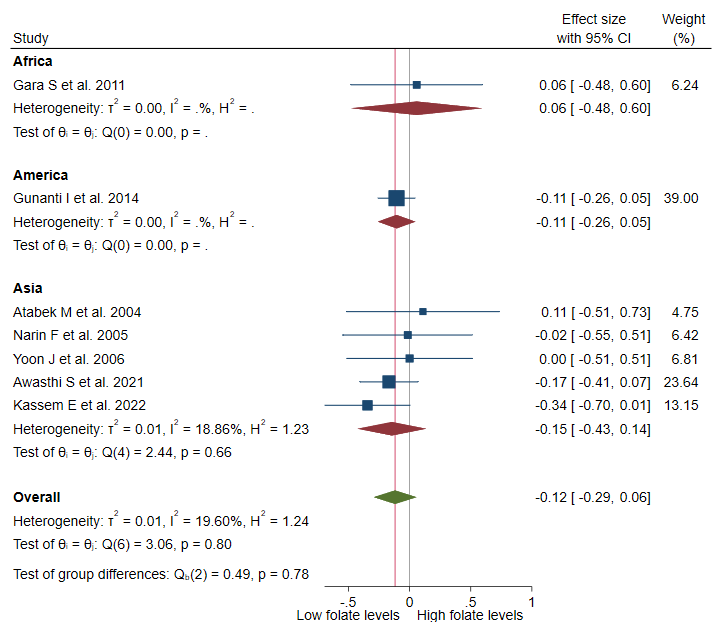
**

**Figure S10. Folate and obesity in children/adolescents: Sensitivity analysis according to risk of bias**


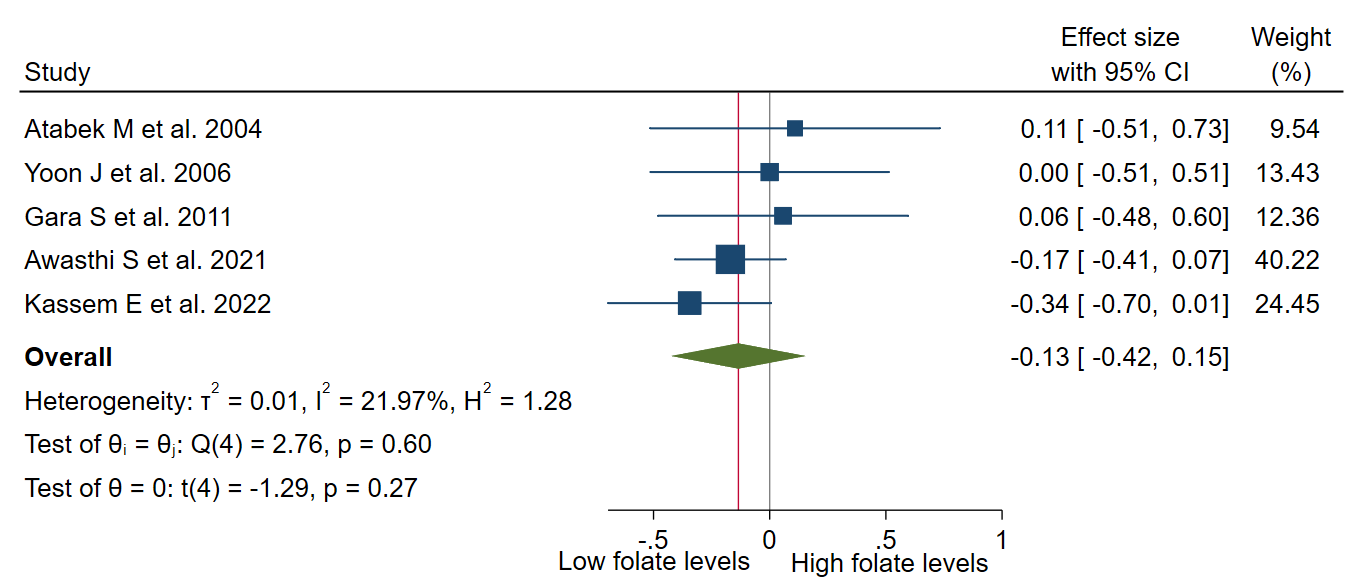


**Figure S11. Homocysteine and obesity in children/adolescents: Subgroup Analysis according to study design**


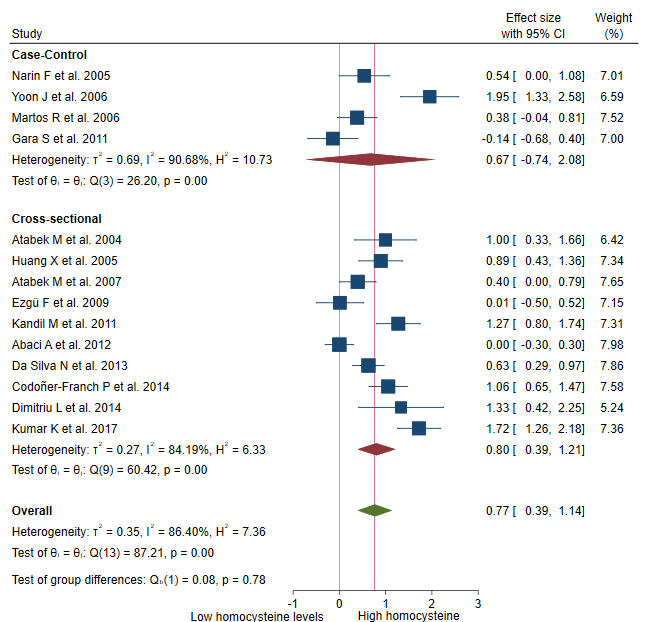


**Figure S12. Homocysteine and obesity in children/adolescents: Subgroup Analysis according to assay method**


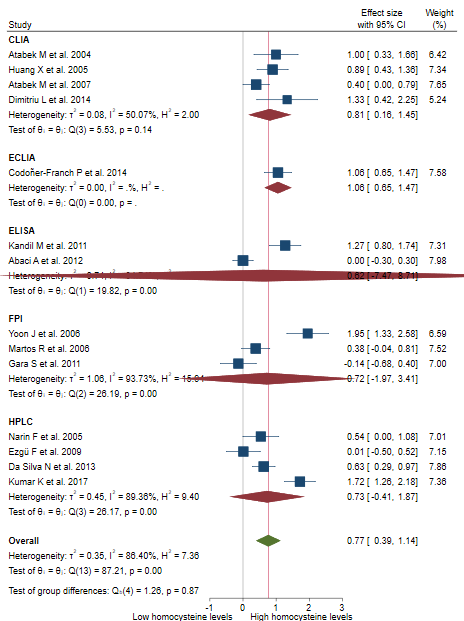


**Figure S13. Homocysteine and obesity in children/adolescents: Subgroup Analysis according to continents**

**
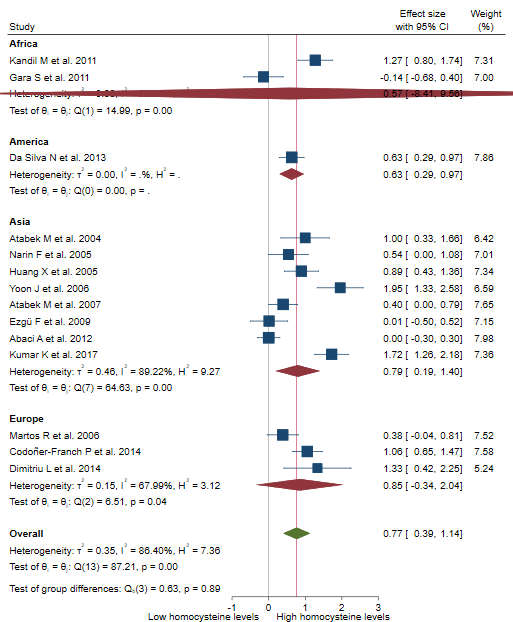
**

**Figure S14. Homocysteine and obesity in children/adolescents: Sensitivity analysis according to risk of bias**


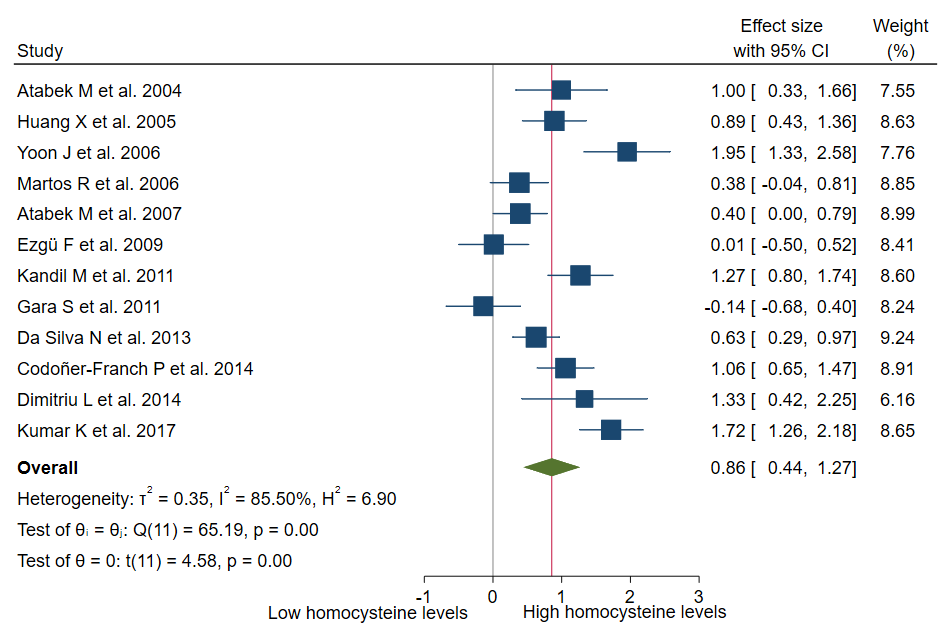


**Figure S15. Homocysteine and obesity in children/adolescents: Sensitivity analysis** **according to obesity definition**

**
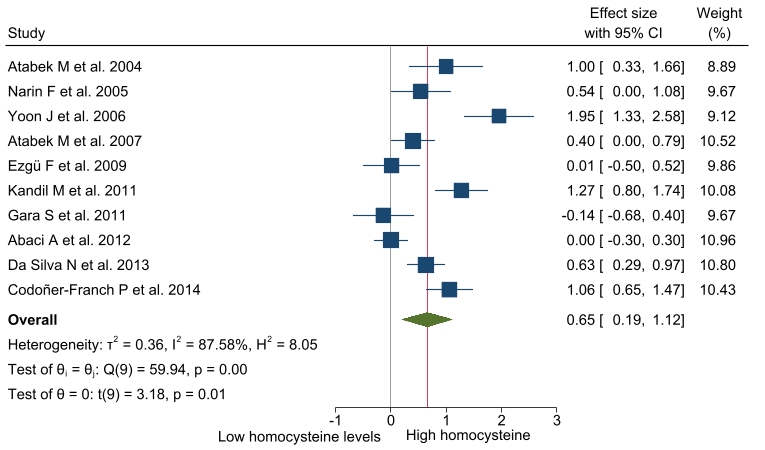
**

**Figure S16. Funnel Plot: Homocysteine and obesity in children/adolescents**


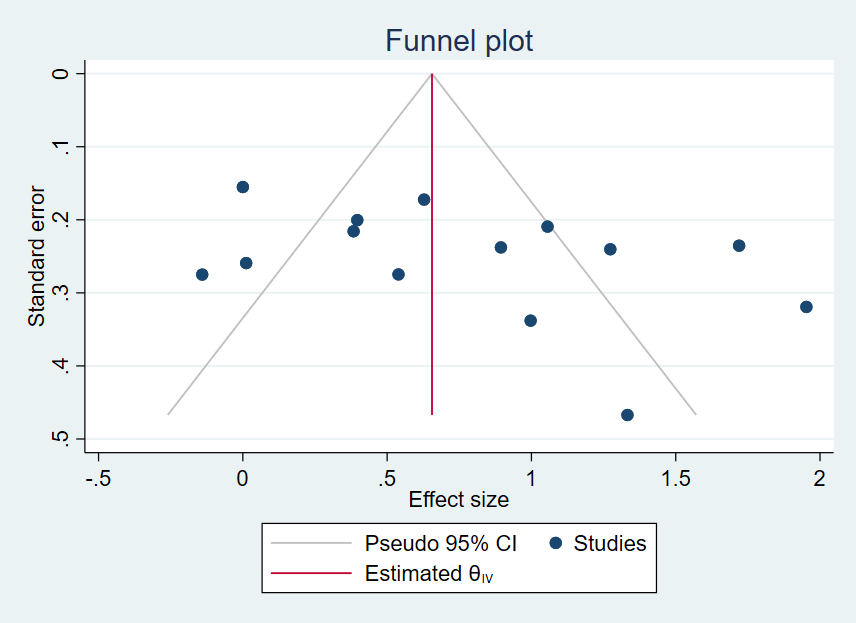

Supplement: Supplementary file 1 [file Data_Sheet_1.docx]
